# In-hospital outcomes and hospital charges after robotic-assisted versus conventional total knee arthroplasty: A 2016–2022 Nationwide Inpatient Sample study

**DOI:** 10.1371/journal.pone.0346031

**Published:** 2026-04-22

**Authors:** David Maman, Yaniv Steinfeld, Yaron Berkovich

**Affiliations:** 1 Technion Israel Institute of Technology, Haifa, Israel; 2 Department of Orthopedics, Carmel Medical Center, Haifa, Israel; Carol Davila University of Medicine and Pharmacy: Universitatea de Medicina si Farmacie Carol Davila din Bucuresti, ROMANIA

## Abstract

**Background:**

Robotic-assisted total knee arthroplasty has been adopted to enhance surgical precision, yet contemporary national evidence on clinical and economic outcomes remains limited.

**Objective:**

To compare in-hospital complications, length of stay, and hospital charges between robotic-assisted and conventional total knee arthroplasty using recent nationwide data.

**Methods:**

We conducted a retrospective cohort study using the Nationwide Inpatient Sample from 2016–2022. Adult elective primary knee arthroplasty admissions were identified; cases with revision procedures or documented coronavirus disease were excluded. Robotic-assisted and conventional procedures were compared after 1:1 propensity score matching on demographics, hospital factors, and comorbidities and year of admission (two cohorts of 173,565 patients each). Outcomes included length of stay, total hospital charges, and major in-hospital complications. Two-sided tests were used with a significance threshold of 0.05.

**Results:**

The proportion of robotic-assisted procedures increased from 0.7% in 2016 to 14.9% in 2022. After matching, robotic-assisted surgery was associated with a shorter mean length of stay (1.9 vs 2.7 days; p < 0.001) and lower rates of several complications. When expressed as relative risks (RR) (risk in conventional TKA divided by risk in robotic-assisted TKA), transfusion (RR 3.7), pneumonia (RR 3.0), pulmonary embolism (RR 2.6), prolonged ventilation (RR 2.1), and deep vein thrombosis (RR 1.8) were all higher in the conventional group (all p < 0.01). Acute kidney injury was marginally more frequent with robotic assistance (relative risk 0.9; p = 0.03). Mean hospital charges were higher for robotic-assisted procedures (US$70,758 vs US$62,618; p < 0.001).

**Conclusions:**

In a large, contemporary national cohort, robotic-assisted total knee arthroplasty was associated with fewer in-hospital complications and shorter hospital stays than conventional surgery, while incurring higher hospital charges. These findings support a potential safety advantage for robotic assistance during the index admission and motivate further study of longer-term clinical and economic outcomes. Levels of Evidence: LEVEL III.

## Introduction

Total knee arthroplasty (TKA) is among the most frequently performed orthopedic procedures in the United States, with annual volumes exceeding one million cases [1]. Despite advances in implant design and perioperative care, up to 20% [2] of patients remain dissatisfied following surgery. This persistent dissatisfaction has fueled interest in surgical innovations that may improve precision, functional outcomes, and complication profiles.

Robotic-assisted TKA (RA-TKA) has emerged as one such innovation, offering enhanced implant positioning accuracy [3], more consistent restoration of limb alignment [4], and potentially improved early function and patient-reported outcomes [5]. Randomized controlled trials and observational studies [6] have reported lower rates of malalignment and soft-tissue trauma [7] with RA-TKA compared to conventional TKA (C-TKA). However, much of the literature has been limited by smaller cohorts, single-center designs, or older datasets that may not reflect current surgical practice.

The adoption of RA-TKA has grown rapidly in recent years, driven by both technological advancements [8] and expanding surgeon familiarity. Evaluating the real-world impact of RA-TKA on perioperative outcomes and healthcare utilization is essential for guiding clinical decision-making and hospital resource allocation. The Nationwide Inpatient Sample (NIS), the largest publicly available all-payer inpatient database in the U.S., provides an opportunity to assess these questions at a national scale [9]. Therefore, this study aimed to analyze the most recent NIS data (2016–2022) to compare perioperative complications, hospital stay, and costs between RA-TKA and C-TKA in a large, nationally representative cohort.

## Materials and methods

### Data source and study design

This retrospective cohort study utilized data from the NIS, the largest publicly available all-payer inpatient care database in the world. Maintained by the Healthcare Cost and Utilization Project (HCUP), the NIS captures a 20% stratified sample of all U.S. hospital discharges and includes over 7 million unweighted hospital stays per year. We analyzed data from 2016 to 2022, with the 2022 dataset representing the most recent and up-to-date version available. This timeframe offers a unique opportunity to study trends and outcomes using the most comprehensive inpatient dataset currently available worldwide. This study is reported in accordance with the Strengthening the Reporting of Observational Studies in Epidemiology (STROBE) guidelines.

### Patient selection

Adult patients (aged ≥18 years) who underwent TKA were identified using ICD-10-PCS procedure codes specific to knee replacement. We included only elective hospital admissions and excluded non-elective cases, revision arthroplasty, and patients with missing demographic or hospital-level data. To minimize bias from pandemic-related changes in care delivery, all admissions with a documented COVID-19 diagnosis (ICD-10 code U07.1) were excluded from the analysis.

### Exposure definition and outcome measures

The exposure of interest was the use of robotic assistance during primary TKA. Outcomes included (1) temporal adoption trends, (2) baseline demographic and comorbidity differences, (3) clinical outcomes such as length of stay and total hospital charges, and (4) a broad set of postoperative complications, including pulmonary embolism, pneumonia, blood transfusion, AKI, and others. Complications were identified using ICD-10-CM diagnosis codes.

### Propensity score matching

To reduce selection bias and ensure balanced comparison between groups, we applied 1:1 propensity score matching (PSM) to patients undergoing robotic vs. non-robotic TKA. Matching was performed using logistic regression with a nearest-neighbor algorithm and a caliper width of 0.01, without replacement.

The matching process yielded two cohorts of 173,565 patients each, for a total of 347,130 matched cases.

Propensity scores were calculated based on a comprehensive set of baseline characteristics, including:

1 **Demographics**

- Age
- Sex
- Race
- Primary payer (Medicare, Medicaid, private insurance, self-pay, other)

2 **Hospital Characteristics**

- Bed size (small, medium, large)
- Hospital location and teaching status (urban teaching, urban non-teaching, rural)
- Hospital region (Northeast, Midwest, South, West)

3 **Admission Characteristics**

- Weekend vs. weekday admission

4 **Comorbidities**

- Hypertension
- Dyslipidemia
- Obstructive sleep apnea
- Chronic kidney disease
- Congestive heart failure
- Type 2 diabetes mellitus
- Chronic anemia
- Chronic lung disease
- Obesity
- Osteoporosis
- Parkinson’s disease
- Alzheimer’s disease
- History of myocardial infarction
- History of cerebrovascular accident (stroke)
- Fibromyalgia
- Disorders of the thyroid
- Alcohol use disorder

and year of admission (2016–2022). Balance between matched groups was assessed using standardized mean differences (SMDs). All matched variables demonstrated SMD < 0.1, indicating acceptable balance and successful matching across the full set of covariates.

### Statistical analysis

Descriptive statistics were reported as means with standard deviations for continuous variables and as frequencies with percentages for categorical variables. Continuous variables were assessed for normality using the Shapiro-Wilk test and for homogeneity of variance using Levene’s test. Between-group comparisons were performed using independent-sample t-tests for continuous variables and chi-square tests for categorical variables. A two-tailed P-value of <0.05 was considered statistically significant.

To evaluate postoperative complication rates between robotic and non-robotic TKA, relative risks (RRs) and corresponding 95% confidence intervals (CIs) were calculated for each complication in the propensity score-matched cohort. These RRs were then visualized using a forest plot, where each dot represents the RR for a specific complication and horizontal lines indicate the 95% CI range. Relative risks (RRs) were calculated as the risk in C-TKA divided by the risk in RA-TKA.

The NIS is a stratified, cluster-sampled database of U.S. hospital discharges. All analyses incorporated HCUP-provided discharge weights to generate national estimates. We describe the NIS survey design features to facilitate reproducibility and interpretation of national estimates. Inferential statistics, including P values and 95% confidence intervals, were calculated using survey-appropriate methods to ensure valid standard error estimation.

Complications with P < 0.01 were included in the forest plot to emphasize statistically robust differences. The red reference line at RR = 1 indicated no difference between groups; RRs greater than 1 reflected elevated risk in the non-robotic group, while RRs less than 1 favored the robotic group. Outliers exceeding three standard deviations from the mean were inspected and retained unless attributable to data entry errors. All analyses were two-sided. All statistical analyses and graphical visualizations were conducted using IBM SPSS Statistics Version 26.0 (IBM Corp., Armonk, NY) and MATLAB Version R2024a (MathWorks Inc., Natick, MA).

### Ethical aspects

This study analyzed publicly available, de-identified hospital discharge data from the HCUP NIS. According to institutional and national regulations, this secondary data analysis does not constitute human-subjects research. The protocol was reviewed and granted exempt status by the Carmel Medical Center Institutional Review Board (Haifa, Israel). All NIS records are fully de-identified in compliance with the U.S. Health Insurance Portability and Accountability Act (HIPAA); therefore, informed consent was not required.

## Results

**Fig 1:**
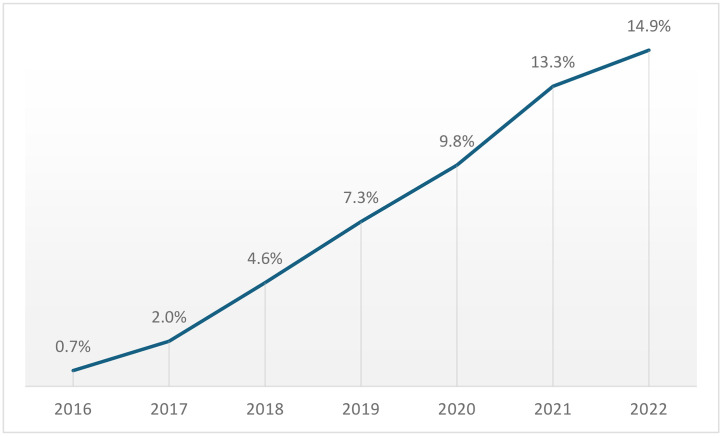
Annual Proportion of Robotic-Assisted Total Knee Arthroplasty Procedures Among All TKA Cases (2016-2022).

As illustrated in Fig 1, the proportion of robotic TKA cases rose from 0.7% in 2016 to 14.9% in 2022 (P < 0.01).

### Demographic and comorbidity differences between robotic and non-robotic TKA patients

As shown in Table 1, robotic-assisted TKA accounted for 5.1% of all procedures, compared to 94.9% performed using conventional methods. Patients undergoing robotic surgery were of similar age to those receiving non-robotic TKA (67.0 vs. 66.9 years, p = 0.01) but included a slightly lower proportion of female patients (59.6% vs. 61.6%, P < 0.01).

**Table 1:** Baseline Demographics and Comorbidities in Robotic vs. Non-Robotic Total Knee Arthroplasty Patients.

Parameter	Non-Robotic Surgery	Robotic Surgery	Significance
Total Surgeries	3,251,564 (94.9%)	173,565 (5.1%)	–
Average Age (y)	66.9	67.0	P = 0.01
Female (%)	61.6	59.6	P < 0.01
Admission day is a weekend (%)	0.6	1.1	P < 0.01
Hypertension (%)	59.1	57.7	P < 0.01
Dyslipidemia (%)	47.3	48	P < 0.01
Obstructive Sleep Apnea (%)	13.6	14.8	P < 0.01
Chronic Anemia (%)	5.8	5.3	P < 0.01
Alcohol Abuse (%)	0.9	0.7	P < 0.01
Osteoporosis (%)	4.2	3.8	P < 0.01
Parkinson Disease (%)	0.6	0.6	P = 0.85
Alzheimer Disease (%)	0.2	0.2	P = 0.12
Chronic Kidney Disease (%)	7.6	7.2	P < 0.01
Type 2 Diabetes (%)	22	20.7	P < 0.01
Congestive Heart Failure (%)	1.3	1.3	P = 0.12
Chronic Lung Disease (%)	6.2	5.5	P < 0.01
Disorders of Thyroid (%)	17.9	17.7	P = 0.02
Fibromyalgia (%)	2.8	2.6	P < 0.01
History of Myocardial Infarction (%)	3.3	3.2	P = 0.38
History of Cerebrovascular Accident (%)	4.2	4.2	P = 0.95
Obesity (%)	31.8	32.4	P < 0.01

Continuous variables were compared using independent-sample t tests. Categorical variables were compared using Pearson χ² tests. All analyses incorporated survey weights.

Several comorbidities demonstrated statistically significant differences. Robotic TKA patients had slightly lower rates of hypertension (57.7% vs. 59.1%), chronic anemia (5.3% vs. 5.8%), alcohol abuse (0.7% vs. 0.9%), and chronic lung disease (5.5% vs. 6.2%), while rates of obstructive sleep apnea (14.8% vs. 13.6%), dyslipidemia (48.0% vs. 47.3%), and obesity (32.4% vs. 31.8%) were marginally higher. No significant differences were observed in the prevalence of Parkinson’s disease, Alzheimer’s disease, congestive heart failure, or prior history of myocardial infarction or stroke.

### Propensity score-matched comparison of robotic and conventional total knee arthroplasty patients

Table 2 summarizes the baseline characteristics of robotic and non-robotic TKA patients after 1:1 propensity score matching, yielding two balanced cohorts of 173,565 patients each. Following matching, no statistically significant differences were observed between the groups in terms of age, sex, weekend admissions, or comorbid conditions. Key variables such as hypertension, type 2 diabetes, dyslipidemia, obstructive sleep apnea, chronic kidney disease, and obesity were evenly distributed, with p-values >0.05 across all comparisons. This confirms the effectiveness of the matching process in minimizing selection bias. All matched covariates achieved standardized mean differences (SMD) <0.10.

**Table 2:** Baseline Demographics and Comorbidities in Propensity Score-Matched Robotic vs. Non-Robotic TKA Patients.

Parameter	Non-Robotic Surgery	Robotic Surgery	Significance
Total Surgeries	173,565 (50%)	173,565 (50%)	–
Average Age (y)	67.0	67.0	P = 0.90
Female (%)	59.6	59.6	P = 0.64
Admission day is a weekend (%)	1.1	1.1	P = 0.74
Hypertension (%)	57.6	57.7	P = 0.97
Dyslipidemia (%)	48	48	P = 0.87
Obstructive Sleep Apnea (%)	14.6	14.8	P = 0.68
Chronic Anemia (%)	5.3	5.3	P = 0.96
Alcohol Abuse (%)	0.7	0.7	P = 1
Osteoporosis (%)	3.8	3.8	P = 0.38
Parkinson Disease (%)	0.6	0.6	P = 0.72
Alzheimer Disease (%)	0.2	0.2	P = 0.80
Chronic Kidney Disease (%)	7.2	7.2	P = 0.68
Type 2 Diabetes (%)	20.7	20.7	P = 0.99
Congestive Heart Failure (%)	1.3	1.3	P = 0.41
Chronic Lung Disease (%)	5.7	5.5	P = 0.51
Disorders of Thyroid (%)	18	17.7	P = 0.18
Fibromyalgia (%)	2.7	2.6	P = 0.24
History of Myocardial Infarction (%)	3.4	3.2	P = 0.43
History of Cerebrovascular Accident (%)	4.2	4.2	P = 0.90
Obesity (%)	32.3	32.4	P = 0.74

Continuous variables were compared using independent-sample t tests. Categorical variables were compared using Pearson χ² tests. All analyses incorporated survey weights.

### Propensity score-matched comparison of length of stay, hospital charges, and complication burden in robotic vs. non-robotic TKA

In the propensity score-matched analysis shown in Table 3, robotic TKA was associated with a shorter hospital stay compared to non-robotic surgery (mean 1.9 vs. 2.7 days, p < 0.001). Total hospital charges were significantly higher in the robotic group, with an average of $70,758 compared to $62,618 in the non-robotic group (p < 0.001). The proportion of patients experiencing any in-hospital complication was significantly lower in the robotic group (20.0% vs 27.0%; P < 0.001).

**Table 3:** Comparison of Length of Stay, Hospital Charges, and Complication Rates in Propensity Score-Matched Robotic vs. Non-Robotic TKA Patients.

	Non-Robotic Surgery	Robotic Surgery	Significance
Length of stay mean in days	2.7 (Std. deviation 1.4)	1.9 (Std. deviation 1.7)	P < 0.001
Total charges mean in $	62,618 (Std. deviation 39,478)	70,758 (Std. deviation 46,435)	P < 0.001
Any in-hospital complication (%)	27.0%	20.0%	P < 0.001

Continuous variables were compared using independent-sample t tests. Categorical variables were compared using Pearson χ² tests. All analyses incorporated survey weights.

### Forest plot showing elevated risk of postoperative complications in non-robotic compared to robotic TKA in propensity score-matched groups

**Fig 2:**
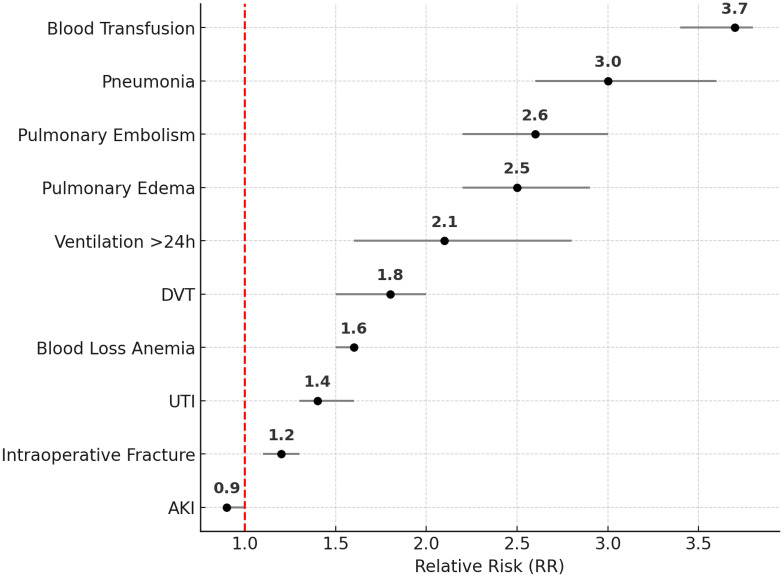
Forest Plot Showing Elevated Risk of Postoperative Complications in Non-Robotic Compared to Robotic TKA in Propensity Score-Matched Groups.

Fig 2 displays a forest plot illustrating the relative risk (RR) and 95% confidence intervals for major postoperative complications in non-robotic versus robotic total TKA, based on propensity score-matched groups. Each dot represents the RR, and the horizontal line shows the 95% CI range.

Non-robotic TKA was associated with significantly higher risks for nearly all complications, including:

- Blood transfusion (RR 3.7, 95% CI 3.4–3.8, P < 0.01)
- Pneumonia (RR 3.0, 95% CI 2.6–3.6, P < 0.01)
- Pulmonary embolism (RR 2.6, 95% CI 2.2–3.0, P < 0.01)
- Pulmonary edema (RR 2.5, 95% CI 2.2–2.9, P < 0.01)
- Ventilation for more than 24 hours (RR 2.1, 95% CI 1.6–2.8, P < 0.01)
- DVT (RR 1.8, 95% CI 1.5–2.0, P < 0.01)
- Blood loss anemia (RR 1.6, 95% CI 1.5–1.6, P < 0.01)
- UTI (RR 1.4, 95% CI 1.3–1.6, P < 0.01)
- Intraoperative fracture (RR 1.2, 95% CI 1.1–1.3, P < 0.01)

For AKI, the RR was 0.9 (95% CI 0.9–1.0; P = 0.03), indicating a slightly higher rate in the RA-TKA group compared with C-TKA. Sepsis (RR 0.1, P = 0.73) was excluded from the plot due to lack of statistical significance.

## Discussion

### Main findings

This updated national analysis of over 3.4 million TKA from 2016 to 2022 shows that RA-TKA is associated with significantly reduced in-hospital complication rates and shorter LOS compared to C-TKA, despite slightly higher hospital charges. These findings build upon earlier reports [10,11] by incorporating a larger matched cohort, a longer study period, exclusion of COVID-19-related admissions, and the most recent available data, thereby enhancing both statistical power and contemporary relevance.

Across nearly all evaluated complications, RA-TKA was associated with lower in-hospital complication rates compared with C-TKA. Relative risk reductions were most pronounced for blood transfusion, pneumonia, pulmonary embolism, and prolonged ventilation. These differences remained significant after rigorous propensity score matching, suggesting that the observed advantages may not be solely attributable to differences in case mix or hospital characteristics [12,13].

### Clinical implications

THE consistent reduction in acute complications suggests that RA-TKA may be associated with lower in-hospital complication rates. Lower transfusion rates likely reflect reduced intraoperative blood loss due to the avoidance of intramedullary guides and the precision of bone resections [14,5]. Decreased rates of pneumonia, pulmonary embolism, and prolonged ventilation may indicate a smoother postoperative course, potentially facilitated by earlier mobilization and reduced systemic inflammation [15,16]. Although the absolute risk reduction for each complication is small, the cumulative benefit across a high-volume national population is meaningful.

Shorter LOS further strengthens the clinical value of RA-TKA. Even a reduction of 0.5–1 day has important implications for bed availability, discharge planning, and overall hospital throughput. These findings align with prior research showing that RA-TKA patients achieve functional milestones earlier and have a greater likelihood of being discharged home [17,18], making this technology well suited for fast-track and outpatient arthroplasty pathways.

### Economic considerations

While RA-TKA is associated with higher upfront hospital charges, these figures should be interpreted in the context of bundled payments and value-based care models [19]. Although this analysis only assessed index hospitalization charges, previous studies suggest that overall episode-of-care costs may be lower for RA-TKA due to reduced post-acute care utilization and fewer readmissions [20,21]. As robotic platforms become more widely adopted and surgeons progress along the learning curve, per-case costs may decline due to improved efficiency and economies of scale [8,22].

Furthermore, potential improvements in patient-reported outcome measures, implant longevity, and revision rates-although not captured here-may contribute to downstream cost savings [23,24], supporting the economic rationale for RA-TKA in appropriately selected patients.

### Limitations

This study has several limitations. As with all administrative database analyses, accuracy depends on proper ICD-10 coding, and misclassification is possible [25,26]. Although propensity score matching was used to adjust for observed patient and hospital characteristics, residual confounding from unmeasured variables such as surgeon experience, operative time, and rehabilitation protocols cannot be excluded.

The Nationwide Inpatient Sample only captures in-hospital events, so long-term outcomes, readmissions, and patient-reported measures could not be assessed [27]. Hospital charges do not equate to actual costs or reimbursements and vary by institution and region [19]. Finally, outpatient TKAs, which have become increasingly common in recent years, are not included in this dataset. If lower-risk C-TKA patients were preferentially treated in outpatient settings, inpatient C-TKA complication rates could be overestimated.

Although matching included year of admission to mitigate secular differences in hospital charges, we did not inflation-adjust charges across study years; therefore, reported values represent nominal hospital charges and should not be interpreted as inflation-corrected costs. The NIS does not provide information on radiographic osteoarthritis severity, limb alignment, surgical approach, tourniquet use, implant constraint level, robotic platform type, operative time, discharge criteria, or surgeon experience. These factors may influence complication rates and resource utilization and could not be directly adjusted for in this administrative dataset.

Although the matched cohort was large, the relatively small proportion of robotic cases (approximately 5%) limited subgroup analyses and may not fully reflect outcomes in low-volume centers. Coding inaccuracies and residual confounding remain possible despite careful matching. Additionally, outpatient arthroplasties and long-term results were not captured, which may underestimate early discharge trends and sustained benefits.

### Future directions

Future work should assess the long-term clinical and economic value of RA-TKA, ideally through prospective multicenter studies or national joint registries that incorporate PROMs, revision rates, and 90-day outcomes. Identifying patient subgroups such as those with severe deformities, obesity, or complex anatomy that derive the greatest benefit from robotic assistance could inform selective use strategies, maximizing both clinical outcomes and cost-effectiveness.

## Conclusion

This large, contemporary national analysis demonstrates that robotic-assisted total knee arthroplasty was associated with lower in-hospital complication rates and shorter length of stay compared with conventional TKA, while hospital charges were higher. These findings reflect differences observed during the index hospitalization and should be interpreted within the limitations of administrative data.

## Abbreviations

LOS: Length of stay
AKI: Acute kidney injury
DVT: Deep vein thrombosis
UTI: Urinary tract infection
RR: Relative risk
CI: Confidence interval
